# Essential Oil-Based Nanoparticles as Antimicrobial Agents in the Food Industry

**DOI:** 10.3390/microorganisms10081504

**Published:** 2022-07-26

**Authors:** Micaela Guidotti-Takeuchi, Lígia Nunes de Morais Ribeiro, Fernanda Aparecida Longato dos Santos, Daise Aparecida Rossi, Flávia Della Lucia, Roberta Torres de Melo

**Affiliations:** 1 School of Veterinary Medicine, Federal University of Uberlandia, Uberlandia 38402-018, Brazil; micaelaguidotti@gmail.com (M.G.-T.); fe.longato@hotmail.com (F.A.L.d.S.); daise.rossi@ufu.br (D.A.R.); roberta-melo@hotmail.com (R.T.d.M.); 2 Institute of Biotechnology, Federal University of Uberlandia, Uberlandia 38405-320, Brazil; 3 Nutrition Faculty, Federal University of Alfenas, Alfenas 37130-001, Brazil; dellaluciaf@gmail.com

**Keywords:** food science, nanotechnology, pathogens

## Abstract

The use of essential oils (EO) loaded with nanoparticles is the most promising alternative to increase food quality and safety. Interesting works describe the antimicrobial properties of EO for pathogen control in natural and processed foods for human health and animal production, also contributing to sustainability. Their association with different nanosystems allows novel developments in the micronutrition, health promotion, and pathogen control fields, preventing the aggravation of bacterial microevolution and combating antibiotic resistance. Benefits to the environment are also provided, as they are biodegradable and biocompatible. However, such compounds have some physicochemical properties that prevent commercial use. This review focuses on recent developments in antimicrobial EO-based nanoparticles and their application in different food matrices.

## 1. Introduction

Recently, the interest in “green food products” based on natural compounds and minimal processing of edible products [1,2] has gained attention in determining products’ acceptance [3]. These characteristics are crucial factors that reflect the current trends and economic growth in this sector [4], which is estimated to exceed USD 13 billion by 2024, according to reports from Global Market Insights, Inc. (Selbyville, DE, USA) [5]. From this point of view, researchers and the food industry (FI) have been looking for sustainable alternatives, such as the use of essential oils (EOs), to replace synthetic additives [6], such as sorbic, benzoic, propionic acids, and sodium diacetate preservatives [7].

Despite that the microbiological control of contamination in fresh or processed foods is a topic of great interest, this is still a challenge for safe food-based systems developments. There are some pathogens that can cause food spoilage and affect nutritional and sensory qualities, as well as causing foodborne infections and toxicosis. Food contaminated with pathogenic microorganisms causes food spoilage, with consequent economic losses and undesirable effects on product quality and safety [8]. Recently, a report by the Centers for Disease Control and Prevention (CDC) (2021) demonstrated an increase of foodborne diseases related to microbial contamination during the periods 2004–2008 and 2015–2016, by nontyphoidal *Salmonella*, with around 159,000 and 222,000 infections, respectively [9].

Resistance modeling studies estimate an increase in resistance for priority antibiotic–bacterial combinations of 17–31% between 2000 and 2030 for 52 countries, including hotspots related to Brazil [10]. With this current scenario, a thorough analysis of changes and health impacts will help to establish goals and priorities for infection prevention [9]. More efforts in differentiated approaches to ensure and maintain food safety are urgently needed.

The EOs are plant-derived compounds with complex aromatic structures and high volatility. The volatilomes [11] are presented as the total fraction of strong-smelling molecules produced in specialized plant cells (oil cells, ducts, or glands) [12]. They are secondary plant metabolites, which play essential roles in the dynamics of plants with their habitat through protection against pathogens and attraction of polarizers [13]. They can be extracted from all parts of plants (from leaves to roots) by using different methodologies. The presence, yield, and chemotype of EOs are influenced by many climatic and plant nutritional factors, as well as stress conditions. For commercial EO production (where increases in the yield are desirable), breeding and selection programs are implemented as a measure to foster specific compositions [14]. The structural profiles of commercial EOs have been previously provided by gas chromatography and mass spectrometry analysis [8,15,16]. In general, their molecular structure reveals two or three major compounds, corresponding to more than 20% of the full molecule, usually responsible for their biological properties [17,18,19].

The EO bioactive compounds present simultaneous antibacterial, antifungal, antiprotozoal, and antiviral properties. Synergistically, the association of Eos with conventional antimicrobials can result in the improvement of the antimicrobial activity, especially useful for prevention and control programs [20]. These plants also comprise an important part of the human diet, and are used to enhance the flavor, color, and aroma of foods [21], potentially providing safer natural preservatives alternatives [21,22] over synthetic chemical preservatives [23]. In addition, phytogenic additives are vegetal compounds or ingredients incorporated in animal feed that aid in the performance and health of animals and, consequently, to the quality of animal and plant-derived foods [24,25].

**Figure 1:**
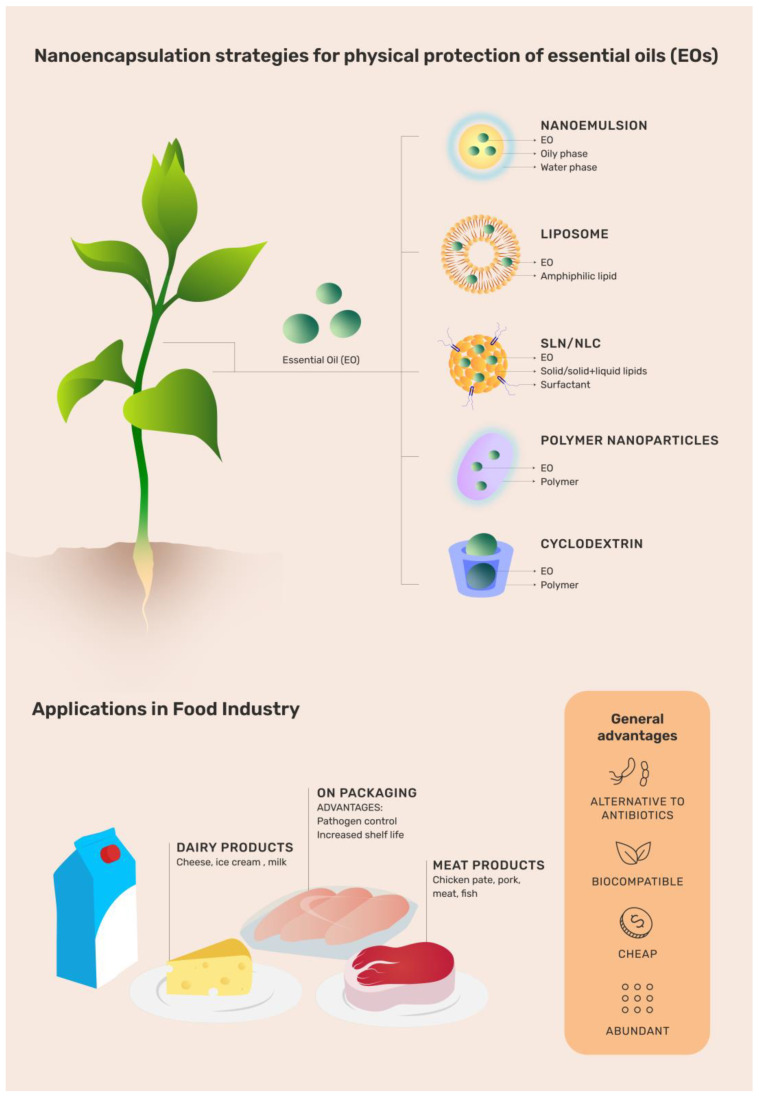
Illustrative chart regarding the use and advantages of different organic nanoparticles loading essential oils (EOs) throughout the food production process. EO: essential oil; SLN: solid lipid nanoparticle; NLC: nanostructured lipid carriers.

However, EOs show a set of undesirable physiochemical properties that prevent their widespread use. They are highly volatile, light-sensitive [26], can produce off-flavors, modify the color of food products [27], interfere with food texture, and have lower bioavailability [28]. In this sense, some nanotechnological approaches are currently applied to overcome these disadvantages without affecting the therapeutic properties. The nanoencapsulation of EOs by different nanocarriers (Figure 1), mainly those with affinity for hydrophobic molecules such as liposomes, nanoemulsions, solid lipid nanoparticles (SLN), nanostructured lipid carriers (NLC), polymer nanocapsules, cyclodextrins, and chitosan-based system (CHT), among others, is a promising alternative for advances in the preservation and microbial control (Table 1) of food products [29,30,31]. Moreover, the different nanocarriers designed for food applications have to be biocompatible, biodegradable, scaled up, and classified as GRAS (generally recognized as safe) [28], which requires technical evidence of safety [32].

This paper provides an overview of EO-related nanosystems with application for different food matrices. The selection criteria of data were focused on the robust works that provided structural characterization of nanoparticles, physicochemical stability, and efficacy assays against food-related pathogens. The resulting data compilation is available in Table 1, containing the composition, types of bioactive compounds, and bactericidal activity against different strains of interest in the FI, as well as the structural properties of the developed nanosystems.

## 2. Production Chain in the Food Industry (FI)

All steps of the food production and processing system (farm to fork) are prone to microbial contamination, with direct consequences to the final consumer, causing different foodborne diseases. Therefore, FIs have a huge challenge in searching new strategies for pathogen control. The misuse of antimicrobials has resulted in a selection of bacteria that are more tolerant and resistant to chemical agents, causing risks to human health, due to ineffective therapeutic efforts [33]. In addition, consumers are concerned about the adverse effects of synthetic components used in food [34]. Regulatory issues related to banning the use of these compounds in animal production [35] are in line with the trend towards consuming foods treated with safe and natural antimicrobials [36].

Antimicrobial resistance has been known since the discovery of antibiotics. The continuous and rapid evolution of microorganisms overcome the effect of antimicrobial agents. Therefore, the drug resistance is a major concern, as human and animal health, food production, and environment are interconnected [37]. Compared to traditional antimicrobial drugs, EO can reach different microbial targets [38], such as binding bacterial proteins and lowering pH [39] that improve the therapeutic effect against multidrug-resistant bacteria present in food [40], such as *Klebsiella pneumoniae*, *Campylobacter jejuni*, *Staphylococcus aureus*, *Pseudomonas aeruginosa*, *Salmonella* spp., *Shigella* sp., *Enterococcus* sp., and *Escherichia coli* [41], and can be used from animal production to the finished product.

Food-grade nanoparticulate products are already available on the market and include nano-additives and nano-ingredients made from ω-3 fatty acids, incorporated into yogurts [42] and fruit juices [43]. They have the function of not only enriching the product, but also promoting the sensory acceptability of fish oil [44]. Other products developed by Aquanova^®^ include nanoemulsions loading vitamins. Other nanoencapsulated vitamins (Lypo-Spheric Vitamin, Livon Laboratories, Inc. Company Profile, Henderson, NV, USA) manufactured by LivOn Labs offer protection against their degradation in the gastrointestinal system, increasing the bioavailability [45]. There are also some reports related to the NP application for sensory enhancements, to inhibit flavor loss (infused pure cocoa nanoclusters, RBC Life Sciences ^®^ Inc, Irving, TX, USA) [46], incorporate natural colorants (β-carotene, curcumin; NutraLease ^®^, Ma’ale Adumim, Israel) [47], and offer nanotechnological advantages, such as Nestlé^®^ patented water-in-oil emulsion (10–500 nm), to facilitate microwave thawing of frozen products [48]. The possibilities of applications are wide, including expressive fat reduction (from 16% to 1%) in ice cream production (Unilever ^®^, London, UK) [49] and incorporation of antimicrobial compounds [49] in packaging, and in decontamination of industrial equipment [47], demonstrating the diversity of application in food matrices and a dynamic approach to food and feed quality assurance [33].

**Table 1:** The main properties of nanostructured delivery systems (NDSs) loading different essential oils and their uses in the food industry.

NDS	Composition	Size	EO-Loaded	Target Organism	In Vitro Study	Outcome	Reference
Liposome	Metil-N-Metilantranilato and the sesquiterpene alcohol α -bisabolol.	9.37 ± 4.69 µm.	*Zanthoxylum tingoassuiba.*	*S. aureus,* multidrug-resistant *S. aureus* isolate, and dermatophyte fungi.	Disc diffusion test.	Significant results against Gram-positive bacteria and dermatophyte fungi.	[50]
Liposome	Silver nanoparticles mixed with chitosan.	200 nm.	Laurel.	Coating films for pork packaging. *S. aureus* and *E. coli.*	In cumulative release test.	Higher antimicrobial activity on S. aureus. Increased shelf life and no toxicity.	[51]
Nanoemulsion	Medium-chain triglyceride and Tween 80^®^.	-	Carvacrol.	*Salmonella enterica* Enteritidis and *Escherichia coli* O157:H7 in contaminated broccoli and radish seeds.	Most probable number (MPN).	Reduced emulsion stability implies lower antimicrobial activity in the presence of organic load.	[52]
Nanoemulsion	Oil/water.	110 nm.	*Thymus capitatus.*	Effects on the quality of bacteria-contaminated milk.	Total viability count.	Interaction of pasteurization + EO inhibited bacterial growth and presented best preservation.	[53]
Nanoemulsion	Soy lecithin + medium-chain triglyceride.	146.9 nm.	Oregano.	Effects on count bacterial growth.	Total aerobic bacteria count (log10 UFC/g) by deep inoculation on a plate count agar (PCA).	Decreased bacterial count; extended shelf life with minimal modifications in the sensory properties of hake burger.	[54]
Nanoemulsion	Tween 20 Trans-Cinnamaldehyde.	127 nm	Trans-cinnamaldehyde from cinnamon oil.	*Escherichia coli* O157:H7; *S. typhymurium; S. aureus.*	Disc diffusion test.	Inhibition test against *S.* Typhimurium and *S. aureus* at pure water and watermelon juice.	[55]
Nanoemulsion	PEG-40 hydroxylated castor oil, sorbitan monooleate.	35–55 nm.	Oregano *(Origanum vulgare)*	Prevention and control of bacterial growth (*S. aureus* and *E. coli)* in chicken pâté.	Minimum inhibitory concentration (MIC) and minimum bactericidal concentration (MBC)	Higher antibacterial action for Gram-positive and Gram-negative (MIC value: 0.56; 0.60 mg/mL/ MBC: 0.90; 3.32 mg/mL).	[56]
Nanoemulsion	Alginate and essential oils.	20–190 nm.	Thyme, lemongrass, and sage.	*E. coli.*	Film–bacteria contact.	The antimicrobial activity of the film formed from nanoemulsions with the encapsulated EO’s resulted in improved antimicrobial activity, better transparency, resistance to water vapor and flexibility.	[57]
Nanoemulsion	Medium-chain triacylglycerol.	<200 nm.	Mint.	*S. aureus* and Listeria monocytogenes.	MIC.	The nanoemulsions with EO showed higher antimicrobial activity in addition to maintaining high stability for at least 30 days of storage.	[58]
Nanoemulsion	Whey protein isolate and maltodextrin.	127–314 nm.	Eugenol.	*E. coli* and *L. monocytogenes.*	MIC and minimum bactericidal concentration (MBC).	Nanoparticulate eugenol exhibited greater antimicrobial activity than when dispersed, as well as showing uniform distribution and better solubility in the food system.	[59]
Nanoemulsion	Lauric alginate, essential oil, and Tween 80.	100 nm.	Cinnamon.	*Salmonella enteritidis, E. coli,* and *L. monocytogenes.*	MIC.	The addition of the surfactant resulted in lower antibacterial activity. High growth inhibition of L. monocytogenes when treated with the nanoemulsion	[60]
NLC	Cocoa butter and Tween 80.	150 nm.	Cardamom.	Application in water-based foods (*S. aureus* and *E. coli).*	Broth macrodilution method.	Better activity on *E. coli* versus *S. aureus* (MIC = 275 and 4400 µg/mL, respectively).	[61]
NLC	Lipid matrices, Plantaren 1200^®^ and Pluronic 188^®^.	168.8–202.6 nm.	Olibanum (formulation 6), salvia (formulation 14) and candeia EO (formulation 19).	Evaluation of antimicrobial activity in planktonic and sessile forms against *Campylobacter jejuni.*	Disc diffusion test, minimum inhibitory concentration of free and sessile bacteria forms.	Olibanum formulation: controlled both free and sessile form Growth inhibition zone: 36 mm; free form MIC value: 1.56–2.6 mg/mL and sessile form: 0.78 mg/mL.	[31]
Cationic biopolymer DDS	1.5% *w*/*v* chitosan; 2% (*v*/*v*).	Thickness of the films: 16.5 ± 1.41 and 17.25 ± 2.04 μm chitosan + 2% OPEO.	Orange peel (OPEO).	Improved shelf life/ inhibited the total psychrotrophic bacteria count of fresh shrimps.	Radical scavenging activity, antimicrobial activity, sensory analysis, and melanosis evaluation.	Longer shelf life (15 days). Chitosan with 2% OPEO: highest inhibitory activity for *Bacillus subtilis*; *S. aureus; E. coli*, *P. aeruginosa;* and *C. albicans.*	[62]
Chitosan nanofibers with EO-liposomes	Chitosan (20 mg/mL) EO with 6 mg/mL.	Tea tree liposomes/chitosan: 150 nm and 300 nm.	Tea tree oil (TTO).	*S. enteritidis* and *S.* Typhimurium	CFU counting method.	Reduction around 5 log_10_ of microorganism in chicken meat after 4 days storage.	[63]
Chitosan films	Edible coatings/films.	Review.	Cinnamon, clove, thyme, tea tree, citrus, garlic.	Pathogen’s control: meat and fish.	Disc diffusion method, food systems.	Associated chitosan–EOs resulted in better preservation, higher antioxidant and antimicrobial effect against important food pathogens when compared to chitosan.	[64]
Chitosan NP	200 mg/kg nanoencapsulated.	-	*Cuminum cyminum.*	Alternative to antibiotic growth promoter in broiler chicks.	*In vivo*: broiler growth performance and immune response.	As in-feed growth promoters in poultry production, chitosan plus cuminum EO improved growth performance and increase in lymphocytes counts.	[65]
Chitosan NP	Chitosan and lemongrass essential oil.	175–235 nm.	Lemongrass.	*Escherichia coli*, Staphylococcus aureus, *Listeria monocytogenes*, and *Candida albicans*.	MIC, MBC, and disc diffusion test.	Time and pH dependent on the active ingredient release characterized by 3 stages of release and high antibacterial activity.	[66]
Chitosan NP	Chitosan and lemon essential oil.	4.7 ± 1.2 nm.	Lemongrass.	*Staphylococcus aureus*, *Listeria monocytogenes*, *Shigella dysenteriae,* and *Escherichia coli.*	MIC.	Potentiation of anti-bacterial activity, especially to strains of *Shigella dysenteriae.*	[67]
Chitosan NP	Chitosan and *Zingiber* essential oil officinalis.	198.13–318.26 nm??	*Zingiber officinalis?*	*Salmonella* Typhimurium and *S. aureus*	Disc diffusion test, stability test.	Excellent antibacterial activity, improved stability and solubility of formulations.	[68]
Chitosan NP	Chitosan and clove essential oil.	223–444 nm.	Clove.	*S. aureus* and *Listeria monocytogenes.*	Disc diffusion test.	Higher encapsulation efficiency active and high anti-bacterial activity being efficient in new applications such as active packaging application.	[69]
Hybrid nanofilm	Alginate, cinnamon essential oil and Tween 80.	92.2 nm.	Cinnamon.	*Salmonella* Typhimurium, *Bacillus cereus, Escherichia coli, and S. aureus.*	Disc diffusion test.	High antibacterial effect and possibility of use as antibacterial packaging.	[70]
Chitosan (Film)	Bio-based zein films Tween 80.	102 ± 5.9 nm.	Cinnamon EO (2–4% *w*/*w*).	*Escherichia coli* and *Staphylococcus aureus.*	*Disc diffusion method: zein film nanocomposites.*	Growth was considerably inhibited by the combination with zein + EO films on Gram-positive bacteria.	[71]
Nanogel	Chitosan–caffeic acid.	100 nm.	Cuminum cyminum.	*Aspergillus flavus.*	MIC.	Nanogels with encapsulated EO’s demonstrated greater antimicrobial activity than without encapsulation	[72]
β-cyclodextrin (CD) and polylactic acid and (qual polimero)?	Nanofilm.	CD: 320 nm.	Cinnamon essential oil.	*Escherichia coli* and *Staphylococcus aureus.*	MIC, MBC.	MIC and MBC: 1 mg/mL and 7 mg/mL, respectively (corresponding to CEO concentrations of 11.35 μg/mL and 79.45 μg/mL) applied to active pork packaging.	[73]
Silica nanoparticle in polypropylene film	Film polymer coated with silica nanoparticle and EO.	100 nm (silica NP).	*Pistacia atlantica* (wild pistachio) EO.	*Staphylococcus aureus*, *Salmonella enterica*, *Escherichia coli*, and *Listeria monocytogenes.*	Vapor diffusion method, stability test.	Results with 0.001 g silica nanoparticles have the highest inhibitory effect on counts and the new milk packages reduced the growth of all bacteria tested. Higher shelf life of milk until 35 days.	[74]
Chitosan film and Titanium dioxide NPs (TiO_2_)	Film with TiO_2_ NP.	Film thickness: 0.08 mm.	*Cymbopogon citratus* (lemongrass).	*Enterobacteriaceae, psychrotrophic bacteria, S. aureus,* and *Lactic acid bacteria.*	Plate count agar expressed as logarithms of colony forming units (CFU)/g minced meat, stability test.	Treatments with 1.5% *C. citratus* EO and 1% TiO_2_ increased the shelf life of the minced meat and control of all microorganisms evaluated at refrigeration temperatures to acceptable levels after 10 days of storage.	[75]
Gelatin nanofibers and β-cyclodextrin	Thyme essential oil/β-cyclodextrin ε-polylysine nanoparticles (TCPNs).	-	*Thyme essential oil.*	*Campylobacter jeuni* *A.*	Plate count method.	Thyme essential oil/β-cyclodextrin ε-polylysine nanoparticles (TCPNs) conjugated with gelatin nanofibers showed activity against *C. jejuni* on chicken meat and preservation of sensory characteristics.	[76]

### 2.1. Essential Oils with Antimicrobial Activity in Edible Products

Lately, “green nanotechnology” has gained attention, being a recurrent demand of industry and consumers. Minerals, vitamins, antioxidants, natural dyes, micronutrients, and EOs can be exploited using these systems. The uses of EOs as natural antimicrobials and preservatives in FI is growing [37], also aiming at controlling biofilm formation [77,78] without affecting the intrinsic sensory properties of edible products [79]. Several EOs metabolites possess antimicrobial activity, differing in their composition, location, and mechanisms of action [80]. They can be loaded by different nanoparticles, mainly composed of lipids and/or polymers, and prepared by different methods (supercritical fluid emulsion extraction, spray-dryer, homogenization, emulsification–solvent evaporation, and ultra-sonication) [81,82,83,84].

In general, it is formed an interface between the EO-based nanosystems and the hydrophobic food matrix, resulting in a synergistic complex, increasing the stability and sustaining the EO release specifically to the target site [3]. In this sense, the bacteria cytoplasmic membrane remodeling is among the most sensitive of cell stabilization in response to biochemical events. Such interactions can promote stretching and compression forces that generate important modifications and deconfiguration in their structure, directly related to the shape of the NP and that allows a higher affinity of interaction with the membrane, causing diffuse elongation and its rupture, due to a mechanical process, induced by tension [85]. As a result, an exacerbated permeability process can occur [86] that affects cellular metabolism and ion leakage, destroys genetic material, and inhibits bacterial quorum sensing signaling [87], also promoting the release of microbial cellular contents, leading to the death of the pathogen [88]. Preventive mechanisms against reactive oxygen species, protein oxidation, and mitochondrial alterations complement the cell damage, promoting injury and interruption of microbial development.

It is important to note that other mechanisms of action include access to the periplasm [89], adenosine triphosphate ATP alteration and cellular hyperpolarization [90], modification of the melting point of membrane microenvironment [91], reduction of the membrane protein interaction, interference in flagella formation, and microbial movement [92] and alteration of the fatty acid organization of the membrane [93]. The barrier imposed by the type of cell membrane, especially in Gram-negative species, is almost completely impermeable to the hydrophobic molecules. More studies are still required of the unique components of these oils (location of hydroxyl groups, alkyl groups, type of aromatic rings) in multiple strains of microorganisms, even belonging to the same family. Thus, the EO therapeutic potential is determined by the spectrum of microorganisms it can inhibit [5]. This includes evaluating whether the action of the active compounds is the same [94], which allows incipient promotion, in consideration of a standardized tetrad of action: nano–EO–bacteria–food matrix. Furthermore, EO can also act as a powerful tool to reduce bacterial resistance [95], evidenced by increased research in various food fields, such as fish [96], chicken pâté [56], milk [79], pork [51], and beef [97], revealing the urgent demand for innovation in the field of food safety. In order to make the use of EOs viable at a large scale, it is necessary to include data such as how much demand there will be for the product; whether this will be seasonal or not; check where, exactly, the customers are; what the market outlook is; check the existence and availability of resources; define what taxes will have to be paid; and who will work in the production and management process.

### 2.2. The Drawbacks of Industrial Use of Essential Oils (EOs)

There are relevant EOs limitations that prevent their large use in functional food developments, such as the strong aroma, instability, volatile compounds, sensitivity to processing conditions [98], and controlling to avoid degradation during the processing and consumption stages. Another problem is its storage, which should be kept in closed containers, away from the light and extreme temperatures to avoid oxidation, peroxidation (essential oils—ECHA) [99], and polymorphic rearrangement process [100]. Their effectiveness varies depending on the form of application, the concentration, and storage conditions [79].

Although there are criticisms of the addition of EO in dairy products, reports from the FI attributed several benefits, such as their incorporation as flavoring agents, improving functional properties of chocolate-based products with 0.1% cinnamon EO. However, the use of higher EO concentration (0.3% and 0.5%) will show limited acceptability [101]. This also occurs in food packaging, as the EO strong taste is a limiting factor that can alter the original taste of the food. Encapsulation is possible in order to overcome this adverse condition and widen the variety of food product applications [102]. The application of NPs for food delivery requires that this system should be economically feasible for scale-up of production [103,104], and offers specifics advantages, including improved food matrix compatibility, low-cost storage and simple use, delayed chemical degradation, prolonged release, softening of undesirable off-flavors, and increased antimicrobial potency [105].

## 3. Nanostructured Delivery Systems (NDSs)

The nanostructured delivery systems (NDSs) are molecularly composed of different biomaterials and are processed as several forms in order to interact specifically with the targets.

There are a lot of benefits of the EOs nanoencapsulation approach, such as bioactive protection to the external environment (e.g., products rich in unsaturated lipids can be peroxided over time); sustained release; desirable shelf time; to mask eventual bioactive unpleasant odor and/or flavor; and act as smart packaging with moisture maintenance, protection against pathogens, and monitoring of conditions during distribution [72,81,106]. Different NPs loading EOs have been proposed for pathogen control or incorporated as adjuvants in food-based products [41]. Furthermore, nanoencapsulation presents itself as a method to protect against formation of degraded compounds with toxic derivatives, such as the conversion of safrole (4-allyl-1,2-methylene dioxybenzene), derived from plants of the Lauraceae family (including nutmeg and black pepper) with hepatocarcinogenic effects [81,107].

Thus, the intended application of EO-loaded nanocarriers will direct the selection of the more appropriate nanosystems, excipients, and concentrations, followed by the most viable preparation method. This early planning is essential to ensure a successful preparation, long-term stability, and high rates of encapsulation efficiency of a bioactive [93]. Therefore, the most developed NDS loading EOs for FI uses are discussed in Table 1 below, with key biological results.

### 3.1. Liposomes

Liposomes are spherical vesicles composed of phospholipids spontaneously oriented as lipid bilayers (unilamellar or multilamellar) in aqueous solutions, given by the hydrophobic interactions of nonpolar acyl chains [108]. The amphiphilic nature of lipids is currently exploited to encapsulate food ingredients of different polarities, creating an efficient physical barrier system and protecting bioactives from environmental conditions [109]. The lipid bilayers allow interaction with the hydrophobic encapsulated compounds, being intrinsically related to NDS stability (fluidity, permeability, and polarity, among others), as well as encapsulation efficiency and sustained release profile [110].

In general, the analyzed reports showed that the EO-loaded liposomes provided optimized antimicrobial activity. Sebaaly et al. (2021) developed multilamellar liposomes composed of dipalmitoyl phosphatidylcholine (DPPC) encapsulating EO from *Z. tingoassuiba*, with a mean diameter of 9.37 ± 4.69 µm [111]. The in vitro antimicrobial activity was evaluated by the disc diffusion test. Antimicrobial activity against *Staphylococcus aureus*, *Micrococcus luteus,* and *Streptococcus mutans*, important pathogens related to the food infections, was observed [110].

Another possible application of EO-loaded liposomes for antimicrobial control is incorporation into food packaging, as recently described [51]. In this work, laurel EO was encapsulated by silver nanoparticles and liposomes. The samples were incorporated in pork packaging as preservatives. Liposomes with an average size of 200 nm and a sustained release profile of laurel EO were obtained for both nanosystems, with values around of 29.30% and 11.79% for laurel-EO-loaded liposomes and laurel-based silver nanoparticles, respectively, after 7 days and stored at room temperature. In addition, they presented satisfactory antimicrobial and antioxidant activity, increasing the storage of animal protein to 15 days when kept at 4 °C [51].

Liposomes offer unique function in the transport of hydrophobic and hydrophilic molecules, allowing its applications in different food matrices (dairy, meat, beverages, and confectionery). Furthermore, the encapsulation of two or more EOs in the same systems can be exploited to achieve beneficial synergistic effects.

### 3.2. Nanoemulsions (NEs)

Nanoemulsions (NEs) are stable systems composed of two immiscible phases [112], being processed as water-in-oil or oil-in-water, stabilized by amphiphilic surfactant and co-surfactant. They exhibit excellent structural properties, with particle size between 50–250 nm and a monodisperse distribution [113]. Food-grade NEs containing bioactive components can be used to develop biodegradable packaging films. Promising results were provided of NEs that prevented foodborne diseases. In this sense, carvacrol-based NE was tested in minimal processes as an alternative to calcium hypochlorite use against *Escherichia coli* O157:H7 and *Salmonella* Typhimurium in contaminated broccoli and radish seeds. A system containing 0.4% carvacrol was able to inactivate contamination in low levels (about 2–3 log CFU/g) in radish seeds. The treatment was not effective for broccoli seeds [52].

NEs can also be applied in milk quality control. Jemaa et al. (2018) noted the efficiency of *Thymus capitatus* essential oil in controlling *S. aureus*, which resulted in colony count decreases of 202 × 10^3^ CFU/mL with the pure oil and 132 × 10^3^ CFU/mL when treated with NEs after 24 h of bacterial inoculation [114]. In both treatments, increase in the inhibition of bacterial growth was observed. Such systems can also be used in animal products without changing the physicochemical characteristics of the product, such as chicken pâté, with the incorporation of NE with different amounts of oregano EO (*Origanum vulgare*) [56].

NEs have huge potential for application in FI. Several NE-based products are already commercially available. However, there are still some concerns related to the economic viability of large-scale production, due to the relatively expensive production costs.

### 3.3. Solid Lipid Nanoparticles (SLNs)

SLNs emerged in 1991, firstly described by Professors Müller and Gasco, as an alternative to traditional colloidal systems such as liposomes [115,116]. This system is based on a lipid matrix composed of solid lipid stabilized by a surfactant [30,116]. SLNs have high affinity to load hydrophobic active into the solid matrix [117]. SLNs allow to improve the stability of loaded EOs under various adverse conditions, including food processing, heating, high pressure, drying, UV radiation, and bile salt [118]. Therefore, such a system was designed for applications in food distribution because it has interesting advantages to be used in this sector, such as the effective uptake of fat-soluble assets, sustained release profile, and long-term stability [119,120].

In meat products, SLN loading curcumin was evaluated in the antimicrobial activity assay of hamburger patties inoculated with foodborne pathogens. It was shown that curcumin-based SLN was more effective against Gram-positive than Gram-negative bacteria, stored for 8 days [121]. However, this optimized effect against Gram-positive species is not a rule, since parameters of different lipid compositions of SLN and the cell wall of microorganisms are primary factors [122] that modulate the biological activities of NDS. The activity against Gram-negative bacteria such as *Salmonella typhi* was also demonstrated. SSLNs containing *Eugenia caryophyllata* EO were noted and less effective for Gram-positive bacteria (*S. aureus*), and more effective for Gram-negative bacteria and fungi, in microbial killing [83].

There are a lot of preparation methods and lipid excipients that act as structural bioactive matrices of SLNs, being reproductible at a large scale. Such systems present an emerging field to encapsulate EOs with bactericidal activity [123]. However, in order to allow SLNs application in the FI, more information is still needed regarding their compatibility with the food interface without negatively affecting the physicochemical and organoleptically properties of food products.

### 3.4. Nanostructured Lipid Carriers (NLCs)

NLCs are the second generation of lipid nanoparticles, composed of a lipid matrix formed by a blend between solid and liquid lipids in room temperature, stabilized by a surfactant [115]. They are also considered to be safe and promising nanocarriers for the delivery of hydrophobic compounds, despite their hydrophilicity (colloid liquid systems), which is paramount for the establishment of water-based foods. Therefore, NLCs have industrial relevance as these nanocarriers also combine specific advantages for food uses, such as the ability to be successfully incorporated into transparent and opaque foods and beverages [115]. The encapsulation of EOs by NLCs has been proposed to exhibit higher stability, minimized toxicity, and preserve the therapeutic properties of pristine EOs [124]. EO-loaded NLC formulations have been widely described to increase the antimicrobial activity of EOs and protect them against environmental damage conditions (light, hydrolysis, and evaporation, among others) [31,125].

It is hypothesized that the mechanisms of action of NLCs against bacterial species refer to the nanostructured particles’ sizes together with their surface charges, culminating in microorganism deaths. Cationic NLCs, when in contact with Gram-negative bacteria, will be electrostatically attracted by the membrane, increasing the EO release at the specific target and causing enzymatic reactions that lead to its rupture [126]. In the case of anionic NLCs in contact with Gram-negative bacteria, the hydrogen bonds between the NLC and bacteria membrane will govern their interactions [127].

Keivani Nahr et al. [61] described a versatile strategy to overcome the limitation of applying cardamom EO for food uses. In this sense, cardamom EO was loaded by NLC (with cocoa butter as solid lipid) and applied to water-based foods. Cardamom EO is used to provide flavor to a wide range of processed foods, and it was noticed that such a compound presented in vitro antimicrobial properties against *S. aureus* and *E. coli*, Gram-positive and Gram-negative bacteria, respectively. The average nanoparticle size of NLC was in the range of 118.7–141.7 nm with a polydisperse size distribution (PDI = 0.271–0.468), with excellent negative zeta potential values in the range from −30 to −58 mV [61].

Ribeiro et al. [31] prepared different NLC formulations encapsulating some vegetable and EOs as active molecules against *Campylobacter jejuni* (CJ), in both free and sessile forms, with high interest in public health and food. The authors provided NLC formulations with particle size lower than 250 nm, monodisperse distribution (PDI < 0.170), and shelf life of over a year when stored at 25 °C. Findings were a composition based on ucuuba butter and olibanum EO that presented in vitro minimum inhibitory concentration (MIC) values around 2.60–1.56 mg/mL and 0.78 mg/mL against free and sessile forms of CJ, respectively. Such nanotechnological approach also contributed to an increase in the stability and hydrophilicity of the EO.

These data are relevant and reflect possible advances in the delivery of NLCs with bactericidal effects in FI, since their efficiency was also evaluated on bacteria in a sessile form and whose elimination in these environments is critical. In addition, the use of bioactive excipients to prepare formulations with adequate physicochemical properties and reduce the limitations of pristine EO uses, allied to the large-scale production ability and low productions costs, make both SLN and NLC systems promising NDSs to reach the market.

### 3.5. Polymer Nanocapsules (NCs)

NCs are NDSs formed by a shell (polymer wall) and core (oil core) counterparts that have interesting physicochemical properties for application in food matrices, such as those derived from aliphatic polyester (ε-caprolactone), which are stable in storage and allow a sustained release of loaded actives. Such core–shell structure brings benefits that include the difficulty of contact with external substances and is better suited to lipophilic foods [128].

The encapsulation of EOs by polymer nanocapsules (NC) aims to improve hydrophilicity of insoluble compounds, protection against degradation, prevention of loss of volatile compounds caused by evaporation, and a sustained release. Their antimicrobial activity of NC loading Thymus capitatus EO with high amount of bioactives (43% thymol) was reported [8]. The association of polymers and thymol EO has immediate applicability against important pathogens in the FI (*S. aureus*, *L. monocytogenes*, *B. cereus*, *S. typhi*, *S. dysenteriae,* and *E. coli*).

Different synthetic polymers can also be used together with biopolymers. An NC composed of Balangu seed gum (0.25%), PVA (poly-vinyl alcohol (1%), and Mentha longifolia EO was prepared and showed promising results. This system was tested for EO release profile in various food conditions and showed immediate release in the first 3 min, followed by a continuous and gradual release up to 180 min [129] in aqueous, acidic, and alcoholic media, according to the European Commission regulation 10/2011 EU (10/2011/EC) requirement (EUR-Lex—32011R0010—EN—EUR-Lex, n.d.) [130]. The studies suggest a greater search for compounds that can be incorporated into NCs to verify the action on bacteria commonly related to food products, exploring their action potential.

### 3.6. Cyclodextrins (CDs)

Cyclodextrins (CDs) are cyclic oligosaccharides that have glucose units linked by α-(1,4)-glycosidic bonds, derived from the enzymatic degradation of starch [110], mainly by *B. macerans* [34]. They present a cone-shaped morphology with a hydrophobic cavity and 7.9 Å diameter, and are able to form inclusion complexes with low-aqueous-solubility molecules [50].

The inclusion complexes formed by CDs and EOs improve the EO solubility and physicochemical stability, providing a sustained release of these compounds [131], also acting as physical barrier to protect the EO in procedures such as freezing, thawing, and/or microwaving during food processing [34]. In the FI, they are used to improve flavor and sorption of bitter compounds. Recently, CDs have been noticed as constituents of active packaging of edible products [132]. 

There are several commercially available CD-based products, such as dairy products, food supplements, processed foods, teas, coffees, clarified fruits, sweeteners, honey, and mustard sauce [103]. There are also some reports of industrially and enzymatically obtained CDs with modified starch glucosyltransferase, resulting in a nontoxic product fully processed by the microbiota of the gastrointestinal tract [133]. CD applicability also extends to antimicrobial sachets as food preservatives. Pimenta dioica EO, derived from the *Myrtaceae* family, was effective against *S. aureus* and food spoilage by *B. nivea* mycelia. These sachets, composed of cellulose fiber, allowed the diffusion of the volatile compounds complexed with β-CD and proved to be effective in the control of food pathogens [134].

Freudenberg et al. were pioneers in the preparation of hybrid systems based on CDs linked to chitosan, aiming to reduce the unpleasant taste and odor of fish oil and vegetable oils. There is still need for more efforts to elucidate a wide range of EOs that can form CD inclusion complexes, since their use in active packaging is already studied and applied in the FI, in order to increase the applications ranges [135].

### 3.7. Chitosan-Based Delivery Systems (CHT)

Chitosan (CHT) is a linear cationic polysaccharide formed by (1,4)-amino-deoxy-b-d-glucan chains derived from a deacetylated chitin of crabs and crustaceans, and is biocompatible, biodegradable, and cheap [64]. The use of CHT as a drug carrier has additional advantages as an NDS, such as the protection of the selected active, changes in temperature, and sustained release at the site of interest [136], given by the electrostatic interactions with anionic biological membranes. As a biopolymer, it can be processed as several forms and is abundant, cheap, and biodegradable [137].

CHT application in association with EOs has demonstrated higher antimicrobial activity against foodborne pathogens than biopolymer films and coatings [64]. Specifically, it has demonstrated its advantageous use as an NDS in food preservatives, as it allows a sustained release of active ingredients in the production of food films and coatings [64]. Its inherent antimicrobial activity has been attributed to the electrostatic forces between the cationic amino groups (NH_3_) of CHT and the negatively charged residues of the fatty acids located on the bacterial [138].

CHT processed as films has been shown to delay the release of EOs, also with high concentration of EOs, evaluated in packaging systems [139] for preservation of poultry meat [26], ground beef [140], fresh shrimp [62], and beef [97]. A recent work shows that CHT films functionalized with zein nanoparticles (ZNs) loading EO of *Cinnamodendron dinisii Schwanke* produced stable, homogeneous ZNs with zeta potential values around +30 mV and a polydispersity index less than 0.2, with size in the range of 70–110 nm. CHT NPs loading *Thymus capitatus* EO demonstrated an encapsulation efficiency around 68%, a maximum release profile after 360 min and optimized antimicrobial activity [141]. Other reports described the antimicrobial activity of CHT film through the disc diffusion test. It was demonstrated that the CHT film loading zein-EO nanoparticles increased the antimicrobial activity against *Salmonella typhimurium* and *Shigella flexneri* [142]. In fish, different EO incorporated into gelatin–CHT films were evaluated against different food pathogens and bacteria that lead to decay. Clove-EO-loaded CHT film showed desirable inhibitory action against six targeted microorganisms, including *L. innocua* and *E. coli* [96]. A novel proposed development of two-layer films composed of CHT–PVA carrying *Zataria multiflora* and cinnamon EO was investigated as an active food packaging effective against *S. aureus* and *Aspergillus flavus*. These results were attributed to the increased hydrophilic property of PVA, aiding in the desired release of antimicrobial agents [140].

The nanocarriers mentioned above can also be combined with other natural biomaterial, such as the association of liposomes and chitosan that results in chitosomes hybrid formulations that contribute to liposomal stability. The supramolecular organization of such a hybrid system is provided by the liposome surface functionalization, through the formation of a CHT coating, which can also ensure a targeted delivery and increased antimicrobial efficacy of the EO [111]. The development of a CHT membrane containing tea tree EO loaded by liposomes was reported to have an observed rate of inhibition of *Salmonella* sp. around 99.9%, in an assay with raw chicken packages, after 4 days of treatment, without considerable impact on sensory quality [63]. These data are comparable to irradiation and acidified sodium chloride as chemical treatments of chicken carcasses for reducing the risk of Salmonella in chicken meat processing [143]. Additionally, CHT-based membrane loading Eos in liposomes presents low toxicity and cost.

There are still some factors to be elucidated to expand CHT uses, mainly because its allergen ability, low hydrophilicity, and organoleptic characteristics (fish-like odor and taste) depend on the concentration used. In general, NDS-based CHT seems to be a biocompatible product when combined with other active principles, in addition to its intrinsic antimicrobial capacity.

## 4. Nanopackaging and Smart Control of Food Pathogens

EOs are widely explored for use in food packaging due to their potential antimicrobial activity [98]. Antimicrobial packaging is also able to maintain both the nutritional and sensory quality of food, extending its shelf life. Such approach seems to be an effective alternative to the addition of synthetic preservatives in the packages, as it will be in contact with the food products. Therefore, EOs are currently incorporated into the packages, aiming to decrease the maximum population growth rate and/or prolong the lag phase of the target microorganism by contact inactivation. When used in adequate concentrations, in addition to inhibiting foodborne pathogenic bacteria (*Listeria*, *Salmonella*, *Aeromonas*, *Clostridium botulinum*, *Enterobacter*, and *Staphylococci*) and their toxins, EOs can also improve the sensory properties of the food products [144]. However, the EOs incorporation in active and smart packaging needs to meet crucial safety requirements. For instance, the slow denaturation of EOs from the packaging material to the food surface allows maintaining a high concentration of EOs over a long period of time when compared to the direct addition of EOs in food, the result of which differs by the rapid diffusion of the bioactive with a consequent reduction in antimicrobial activity [145].

The application of nanotechnology in packaging development presents interesting opportunities to exploit the antimicrobial properties of EOs [146]. This strategy has been widely explored, mainly because they act as a physical barrier leading to decreased spoilage and improved inhibition of pathogenic microorganism growth [147]. Nanofilms composed of a thin layer of polymers, created using layer-by-layer deposition techniques, have potential applications in the field of active food packaging. They have been applied directly on the product, whereas non-edible coatings have a protective function specifically on the packaging. Their applicability also extends to surfaces that are in contact with food during processing or to prevent the formation of biofilms [28].

### Final Product: Pure or Nanoencapsulated Essential Oils in Dairy and Meat Products

A supply chain challenge is ensuring that the final product has a distribution of predefined and quality standards, particularly considering the globalization of the food trade and the many current centralized processing facilities that manage a standardized food distribution [148]. The EOs are considered GRAS by the U.S. Food and Drug Administration (FDA) [29,149,150], and are widely used for several purposes, including maintaining safety and improving factors such as microbial reduction and sensory characteristics when applied to meat, chicken, milk, and fruit juice products [56,79,81,151,152].

EOs have different bioactive components, with effects that can be additive or synergistic [153], useful for improving the sensory and nutritional properties of dairy products [27]. Table 1 summarizes different NDS properties with huge interests in the FI, demonstrating their ability to control or eliminate pathogens. The application of nanosystems encapsulating EOs offers several advantages, ensuring their use as promising nanostructured formulations for food application. A synergy between the mixtures of EOs in animal products is observed [79]. Such an approach has shown to be one of the best natural alternatives to pasteurization, to prevent food spoilage by microbes in various food matrices [154], and can be an effective alternative in microbiological control in countries where raw milk consumption is allowed [155,156].

However, it is important to ponder the use of higher concentrations of EOs, due to their sensory effects and the interactions with the physical components of dairy products. The interaction of EO phenolic compounds occurs from the formation of a layer on the surface of some hydrophobic components of proteins and fats in dairy products, which restricts the EO bioavailability. Xue and colleagues noted that the level of fat in dairy products also influenced the inhibition of *L. monocytogenes*. The antimicrobial activity of NE in low-fat milk (5 log CFU/mL) was higher for whole milk (3 log CFU/mL) after 48 h of storage [157]. The effect caused can be explained by the greater action of hydrophobic antimicrobial agents in whole milk, which has higher amounts of fat [158]. The mentioned approaches of incorporating EOs into films and edible coatings, as well as their nanoencapsulation by different nanocarriers, contribute to maintaining EOs’ therapeutic action [158]. These systems minimize the effects of interactions with the food matrix (fat, carbohydrates, or proteins) [27,159,160,161]. Several works have evaluated the addition of EOs to meat and milk products and their derivatives. Other reports evaluated EOs with antimicrobial effect against pathogens of ultra-filtrated soft cheese [162] and ice cream [163]. A decrease in total mesophilic and psychrophilic aerobic bacteria was evaluated. Similarly, NE-based edible antimicrobial coatings prepared from oregano EO extended the shelf life of low-fat cheese [164]. Mint, fennel, and basil EO in yoghurt were able to inhibit the microbial growth [165]. Meat and related products are more prone to spoilage due to the high levels of protein, high water activity, and diverse microorganisms [121]. Pathogenic bacteria in meat are responsible for a significant number of foodborne outbreaks (*Listeria monocytogenes*, *Salmonella* spp., *E. coli* O157:H, and *S. aureus*) and deaths [166].

Several works have reported the promising effects of EOs when incorporated in meat products to control these problems. Pathogen control of meat has also been demonstrated with geraniol- and linalool-EO-loaded NEs. Growth inhibition of *E. coli* K12, *Listeria innocua*, and *Pseudomonas lundensis* in a simulated meat system was observed [167]. Other work described a control of *S. aureus* and Enterobacteriaceae, using chitosan/cinnamon-EO-loaded NE analyzed in meat patties [151]. The application of EO encapsulated by nanosystems should offer a set of advantages that ensures its use as promising nanostructured formulations in FI application. The use of EOs in the food chain is beneficial to inhibit microorganisms contaminations, which justifies its use. It is still necessary to spend more effort to expand the analyses of other EOs loaded by nanosystems still unknown, with novel properties to be explored, increasing the range of alternatives to synthetic preservatives in the FI.

## 5. Conclusions

Alternative control antimicrobials are provided to reduce the occasional food risk from pathogens, increasing the quality and shelf life of products. Specific in vitro and in vitro toxicity tests for application in the FI need to be standardized in order to ensure the safety of these new compounds.

Specifically, regarding the regulatory issues, there are some safety indications on nanoscience and nanotechnology established by the scientific committee of the European Food Safety Agency (2009) for humans [1] (EFSA, 2020 [168]), and in 2011, a guidance of the use of nanomaterials in animal feed by the FDA (Government of the United States, n.d.). According to Brazil, a robust international regulatory framework for the evaluation of nanotechnology-based products is necessary for approval of food and feed containing nanoparticles. Despite the advances already reported, there is no standard protocol for the toxicity testing of nanomaterials that assesses, for instance, the NP excretion mechanisms after digestion [169]. Specifically regarding NDSs for FI applications, it is indispensable to provide safe, stable, and efficient products. It was noted that the complexity of the food matrix (food matrix pH, temperature, and storage time) makes it difficult to investigate NP migration and toxicity. There are still no global regulations for manufacturing, labeling, or in vivo and in vitro toxicity testing, nor for potential environmental harm from nanoparticle residues [170,171].

We emphasized the use of NLCs as a promising alternative when analyzing the aspects of microbiological control and compatibility with foods. It can be prepared with different mixtures between liquid and solid lipids, molecularly planned according to the target bacterium, being resistant to pH variations, food conservation, and stored at room temperature, highlighting desirable aspects for the production of an effective compound that can be produced on a large scale, for use in industries.

The assessment of risk factors is certainly a concern for the subsequent approval of food-based nanostructured products. It is expected that in the near future, FIs will incorporate nanotechnological tools in their production processes, contributing to the effective microbiological control, according to the standards required for each type of food produced. It can increase the specificity of products against target microorganisms for each food.

## Data Availability

Not applicable.
